# Effect of Dispersion Solvent on the Deposition of PVP-Silver Nanoparticles onto DBD Plasma-Treated Polyamide 6,6 Fabric and Its Antimicrobial Efficiency

**DOI:** 10.3390/nano10040607

**Published:** 2020-03-26

**Authors:** Ana I. Ribeiro, Martina Modic, Uros Cvelbar, Gheorghe Dinescu, Bogdana Mitu, Anton Nikiforov, Christophe Leys, Iryna Kuchakova, Mike De Vrieze, Helena P. Felgueiras, António P. Souto, Andrea Zille

**Affiliations:** 1 2C2T - Centro de Ciência e Tecnologia Têxtil, Universidade do Minho, Campus de Azurém, 4800-058 Guimarães, Portugal; afr@2c2t.uminho.pt (A.I.R.); helena.felgueiras@2c2t.uminho.pt (H.P.F.); souto@det.uminho.pt (A.P.S.); 2 Jožef Stefan Institute, Jamova cesta 39, 1000 Ljubljana, Slovenia; martina.modic@ijs.si (M.M.); uros.cvelbar@guest.arnes.si (U.C.); 3 National Institute for Lasers, Plasma and Radiation Physics, Strada Atomiștilor 409, 077125 Măgurele, Romania; dinescug@infim.ro (G.D.); mitub@infim.ro (B.M.); 4 Centexbel Ghent, Technologie Park 7, 9052 Ghent, Belgium; anton.nikiforov@ugent.be (A.N.); Christophe.Leys@UGent.be (C.L.); iryna.kuchakova@ugent.be (I.K.); mike.devrieze@centexbel.be (M.D.V.)

**Keywords:** silver nanoparticles, dielectric barrier discharge, textile, antimicrobial, exhaustion, spray

## Abstract

Polyvinylpyrrolidone-coated silver nanoparticles (PVP-AgNPs) dispersed in ethanol, water and water/alginate were used to functionalize untreated and dielectric barrier discharge (DBD) plasma-treated polyamide 6,6 fabric (PA66). The PVP-AgNPs dispersions were deposited onto PA66 by spray and exhaustion methods. The exhaustion method showed a higher amount of deposited AgNPs. Water and water-alginate dispersions presented similar results. Ethanol amphiphilic character showed more affinity to AgNPs and PA66 fabric, allowing better uniform surface distribution of nanoparticles. Antimicrobial effect in *E. coli* showed good results in all the samples obtained by exhaustion method but using spray method only the DBD plasma treated samples displayed antimicrobial activity (log reduction of 5). Despite the better distribution achieved using ethanol as a solvent, water dispersion samples with DBD plasma treatment displayed better antimicrobial activity against *S. aureus* bacteria in both exhaustion (log reduction of 1.9) and spray (methods log reduction of 1.6) due to the different oxidation states of PA66 surface interacting with PVP-AgNPs, as demonstrated by X-ray Photoelectron Spectroscopy (XPS) analysis. Spray method using the water-suspended PVP-AgNPs onto DBD plasma-treated samples is much faster, less agglomerating and uses 10 times less PVP-AgNPs dispersion than the exhaustion method to obtain an antimicrobial effect in both *S. aureus* and *E. coli*.

## 1. Introduction

Silver nanoparticles (AgNPs) and their composites are some of the most used nanomaterials. AgNPs have improved properties compared with bulk silver allowing a unique action due to their high surface-to-volume ratio [1]. They have been extensively used in consumer products and medical applications as antimicrobial agents. AgNPs display great antimicrobial properties, revealing a positive effect on Gram positive and Gram negative bacteria, virus and fungi, including various drug-resistant strains [2]. Other metal nanoparticles namely copper, zinc and gold are effective against microorganisms, but silver showed to be more reactive and efficient [3,4,5]. Among functionalized biomedical textiles, AgNPs have been applied in wound and burn dressings, bandages, gazes, surgical sutures, and medical staff uniforms. They are used to avoid cross contamination and treat microbial infections, including healthcare-associated infections (HAIs) and the skin and soft tissue infections (SSTIs) [1,6,7]. HAIs and SSTIs are two of the major problems in worldwide hospitals that significantly contribute to the development of antimicrobial resistance [8,9]. Thus, antimicrobial AgNPs-based materials are nowadays important tools for patients and medical staff [10].

The antimicrobial effect of AgNPs is dependent on the morphological and physicochemical properties such as size, shape, surface charge, capping agent, oxidation state, and AgNPs deposition matrix [11,12,13]. In recent years, important advances have been achieved on the clarification of the antimicrobial mechanism of AgNPs. However, the exact mechanism is still not completely understood [14]. For nanoparticles above 10 nm of size, the most solid theories are based on two principles: (i) silver ions are released from AgNPs surface, destroying bacteria cell membranes. In this case, aerobic conditions enhance the oxidation of AgNPs and also the antimicrobial effect; (ii) the oxidative stress generated by the reactive oxygen species (ROS) onto AgNPs surface modifies cells, resulting in cell death [12]. The use of capping agents is extremely useful to prevent nanoparticles agglomeration, undesirable oxidation and control the release of ions from nanoparticles surface. However, it also influences the physicochemical properties of the nanoparticles and could interfere in its interactions with the surrounding solvent or substrate [15]. Capping agents can stabilize a nanoparticle by electrostatic, steric, hydration forces, depletion, and van der Waals forces [16]. The uncharged polyvinylpyrrolidone (PVP) has great affinity for silver due to its nitrogen and oxygen atoms. PVP-AgNPs shows a superior release of silver ions compared with charged coatings due to the higher dissolution rate [13]. In addition to its AgNPs stabilization during the AgNPs synthesis, PVP can also control nanoparticles rate growth and act as a reducing agent [17]. The conventional antimicrobial agents used in textile industry such as metal salts, quaternary ammonium compounds and triclosan are being replaced due to economic, health, environmental, and efficiency issues [18]. In this context, AgNPs application in textile substrates for antimicrobial purposes has earned wide attention. However, some concerns have been raised due to the possible AgNPs toxicity. AgNPs uncontrolled use can be a serious threat to human health and environment [19,20]. The release of these products must be perfectly controlled to maximize the AgNPs potential and minimize the human risks [18,21]. Several methods are used to incorporate AgNPs into fabrics and can be divided by direct AgNPs stabilization onto fabrics (pad-dry-cure, dip-coating, spin coating, electroless deposition, thermo-synthesizing, spraying, sol-gel, microwave-assisted deposition and ultrasound-assisted deposition) or using pretreatments to activate the surface (plasma treatment using different gases, UV irradiation and other physico-chemical fabric functionalization) [22,23,24,25]. Nevertheless, these methods often consume many chemicals, energy and time; require special equipment; and the antimicrobial agents have weak adhesion to the substrate [10]. A proper AgNPs deposition on textiles must ensure an efficient AgNPs deposition and adhesion during all lifetimes of the materials in order to protect people and the environment from unnecessary AgNPs release [26].

Dielectric barrier discharge (DBD) plasma treatment can be used as a pretreatment to activate the surface of the fabrics. This is a dry environmental friendly, easily adapted to industrial scale and low-cost technology since it may be produced at atmospheric pressure conditions without the use of any chemicals or costly gases [27,28]. In surface activation by DBD plasma treatment, the excited particles by the electric field (including atoms, ions, electrons, photons and free radicals) interact with the substrate, which results in chemical and physical changes [29]. New polar functional groups such as carbonyl, carboxyl, ether, amine, and hydroxyl may be introduced, increasing the oxygen and nitrogen content on textiles surface. Atmospheric DBD plasma treatment also increases micro to nano surface roughness that, in combination with the new functional groups, have demonstrated to improve the AgNPs adhesion [29,30].

In this work, PVP-AgNPs dispersions in ethanol, water and water with alginate were deposited by exhaustion at 30 °C and spray methods on untreated and DBD plasma-treated polyamide 6,6 (PA66) fabrics. It was demonstrated by dynamic light scattering (DLS), reflectance spectroscopy, X-ray Photoelectron Spectroscopy (XPS) and Scanning Electron Microscopy (SEM) analyses the influence of the dispersion solvent in the PVP-AgNPs deposition and of the DBD plasma treatment in the modifications of the PA66 surface. The antimicrobial activity against Gram-negative *Escherichia coli* and Gram-positive *Staphylococcus aureus* bacteria were also assessed.

## 2. Experimental

### 2.1. Materials

Commercial Jersey polyamide 6,6 fabric (PA66) with weight per unit area of 240 g·m^−2^ was supplied for this study. The knitted fabric was pre-washed with 1 g·L^−1^ of non-ionic detergent solution at 60 °C for 60 min, then rinsed with distilled water and dried at 40 °C to minimize the contaminations. All the other reagents were analytical grade purchased from Sigma–Aldrich, St. Louis, MO, USA and used without further purification.

### 2.2. Dielectric Barrier Discharge (DBD) Plasma Treatment

The DBD plasma treatment was performed in a semi-industrial prototype machine (Softal GmbH/University of Minho, Guimarães, Portugal) working at room temperature and atmospheric pressure in air, using a system of metal electrode coated with ceramic and counter electrodes coated with silicon with 50 cm effective width, gap distance fixed at 3 mm, and producing the discharge at high voltage 10 kV and low frequency 40 kHz (Figure S5 in Supplementary Materials). The discharge power supplied by the electrodes and the speed may change, with a maximum discharge of 1.5 kW and speed of 60 m·min^−1^. The dosage applied to PA66 fabric was 2.5 kW min·m^−2^. The machine was operated at optimized parameters: 1 kW of power and velocity of 4 m min^−1^. The plasmatic dosage (*D*) was calculated according to the following Equation (1):

(1) $$D=\frac{P\cdot N}{V\cdot W}$$

where, *P* = power (kW); *N* = number of passages; *V* = velocity (m·min^−1^); and *W* = width of treatment (0.5 m) [31].

### 2.3. Preparation of Silver Nanoparticles Dispersions and Its Deposition onto Polyamide 6,6 Fabric

Polyvinylpyrrolidone-coated silver nanoparticles with 20 nm diameter (Sigma–Aldrich, St. Louis, MO, USA) were deposited in 5 × 5 cm^2^ PA66 samples with and without DBD plasma treatment by spray and exhaustion at 30 °C using PVP-AgNPs dispersions (1 mg·mL^−1^) in ethanol, water and water/alginate solution (25 mg·L^−1^). PVP-AgNPs dispersions were ultrasonicated for 30 min in a Branson 3510 ultrasonic bath followed by 30 more min in an Optic Ivymen System CY-500 ultrasonic tip (Ivymen, Barcelona, Spain). The deposition by spray was applied to both sides with the system pressurized at 1.5 bar and maintained at the distance of 5 cm to the substrate. The deposition by exhaustion was performed in a Ahiba laboratory-dyeing machine (Datacolor, Lawrenceville, NJ, USA) at 30 °C for 60 min, 40 rpm with a 1:100 ratio bath. All samples were dried at 30 °C.

### 2.4. Dynamic Light Scattering (DLS) Measurements

The PVP-AgNPs size distribution, polydispersity index (PdI) and zeta potential (ζ) were measured by dynamic light scattering (DLS) and electrophoretic light scattering (ELS) using a Zeta Sizer-Nano (Malvern Instruments, Malvern, UK). Data were collected after 30 scans at a constant temperature of 25 °C. Zeta potential was measured in solution at a moderate electrolytic concentration. The reported results are the average of three measurements without any dilution.

### 2.5. Diffuse Reflectance (%R)

The diffuse reflectance of untreated and DBD plasma-treated PA66 samples with adsorbed PVP-AgNPs were measured using a Spectraflash 600 (Datacolor, Lawrenceville, NJ, USA) spectrophotometer at standard illuminant D65 (LAV/Spec. Excl., d/8, D65/10°). All measurements were performed in triplicate. The data were expressed as the percentage of reflectance after PVP-AgNPs loading in the nanoparticles maximum wavelength absorbance (420 nm) with respect to the untreated and plasma-treated PA66 controls.

### 2.6. X-ray Photoelectron Spectroscopy (XPS)

XPS analyses were performed using a Kratos AXIS Ultra HAS (Kratos Analytical Limited, Manchester, UK), with VISION software (Vision 2, Kratos Analytical Limited, Manchester, UK) for data acquisition and CASAXPS software (casaXPS 2.3.22, Casa Software Ltd., Teignmouth, UK) for data analysis. The analysis was carried out with a monochromatic Al Kα X-ray source (1486.7 eV), operating at 15 kV (150 W), in FAT (Fixed Analyser Transmission) mode, with a pass energy of 40 eV for regions ROI and 80 eV for survey. Data acquisition was performed with a pressure lower than 10^−6^ Pa, and it was used as a charge neutralization system. Spectra have been charge corrected to give the adventitious C1s spectral component (C–C, C–H) a binding energy of 285 eV. High-resolution spectra were collected using an analysis area of ≈1 mm^2^. The peaks were constrained to have equal FWHM (Full Width at Half Maximum) to the main peak. This process has an associated error of ±0.1 eV. Spectra were analysed for elemental composition using CASAXPS software (version 2.3.15). Deconvolution into sub-peaks was performed by least-squares peak analysis software, XPSPEAK version 4.1 (Prof. Raymond W.M. Kwok, Chinese University of Hong Kong), using the Gaussian/Lorenzian sum function and Shirley-type background subtraction. No tailing function was considered in the peak fitting procedure. The components of the various spectra were mainly modelled as symmetrical Gaussian peaks unless a certain degree of Lorentzian shape was necessary for the best fit. The best mixture of Gaussian–Lorentzian components was defined based on the instrument and resolution (pass energy) settings used as well as the natural line width of the specific core hole.

### 2.7. Scanning Electron Microscopy (SEM)

Morphological analyses of PA66 fabrics were carried out with an Ultrahigh resolution Field Emission Gun Scanning Electron Microscopy (FEG-SEM), NOVA 200 Nano SEM, FEI Company (Hillsboro, OR, USA). Secondary electron images were performed with an acceleration voltage at 5 kV. Backscattering Electron Images were realized with an acceleration voltage of 15 kV. Samples were covered with a film of Au–Pd (80–20 weight %) in a high-resolution sputter coater, 208HR Cressington Company (Oxhey, Watford, UK), coupled to a MTM-20 Cressington High Resolution Thickness Controller.

### 2.8. Antimicrobial Analyses

The antibacterial efficacy of the PA66 samples was assessed quantitatively according to the standard shake flask method (ASTM-E2149-01). Both Gram-positive and Gram-negative bacteria were used, respectively, *Staphylococcus aureus* (*S. aureus*, ATCC 6538) and *Escherichia coli* (*E. coli*, ATCC 25922). The experiment was conducted aseptically to ensure the absence of any contamination. Bacteria inoculum were prepared from a single colony and incubated overnight in tryptic soy broth (TSB, Merck) at 37 ºC and 120 rpm. Each test was carried out using an initial concentration of 1.5–3.0 × 10^7^ CFUs/mL in PBS. PA66 samples of 0.05 g weight were incubated in 5 mL of bacteria suspension at 37 °C and 100 rpm. Before contact with samples (0 h) and after contact with samples (24 h), the bacteria were serially diluted, cultured onto tryptic soy agar (TSA, Merck) plates, and further incubated for another 24 h. Quantitative results were obtained by counting the colonies of surviving bacteria on the agar plates. Antimicrobial activity was reported qualitatively in terms of log reduction, calculated as the ratio between the number of surviving bacteria colonies present on the TSA plates, before and after contact with the fabric. The control samples were treated in the same conditions of the other samples, using the solutions without AgNPs. The antimicrobial efficacy of AgNPs in colloidal dispersion was evaluated in a 5 mg/mL colloidal suspension and examined using the inhibition zone test. It was observed that there was no inhibition due to AgNPs agglomeration. All antimicrobial experiments were conducted in triplicate.

## 3. Results and Discussion

### 3.1. Dynamic Light Scattering (DLS)

The average size and polydispersity index (PdI) of the PVP-AgNPs in ethanol, water and water/alginate dispersions were determined by dynamic light scattering (DLS) (Table 1). The size distribution suggests some agglomeration in the PVP-AgNPs dispersions even after the sonication processes. The smallest PVP-AgNPs were observed in the water (188.0 ± 0.7 nm) and water/alginate (156.6 ± 0.5 nm) dispersions. The PVP-AgNPs-ethanol dispersions show higher agglomeration with an average size of 281.0 ± 1.5 nm. The PdI values are in the range of 0.1–0.4, indicating a moderate polydispersity of the PVP-AgNPs distribution. The surface charge of PVP-AgNPs and the physical stability of the colloidal nanodispersions were estimated by surface zeta potential (**ζ**) measurements (Table 1). The **ζ** values express the electrical charge between the moving and stationary layers around colloidal particles [32]. Thus, it is possible to predict PVP-AgNPs stability and their ability to interact with a textile substrate. The **ζ** value of the PVP-AgNPs dispersions can be influenced by the change in pH values and solvent [33,34]. On one hand, PVP-AgNPs dispersed in ethanol showed an increase from neutral pH to pH 8 and moderate negative **ζ** potential (−31.0 ± 1.2 mV). On the other hand, PVP-AgNPs in water displayed an increase from pH 5 to pH 6 and a **ζ** potential near to zero (−1.4 ± 0.1 mV). The addition of alginate in water dispersion increased the pH value to 7 and stabilized the dispersion increasing the **ζ** potential to moderate negative values (−33.8 ± 1.5 mV).

### 3.2. Reflectance

**Figure 1:**
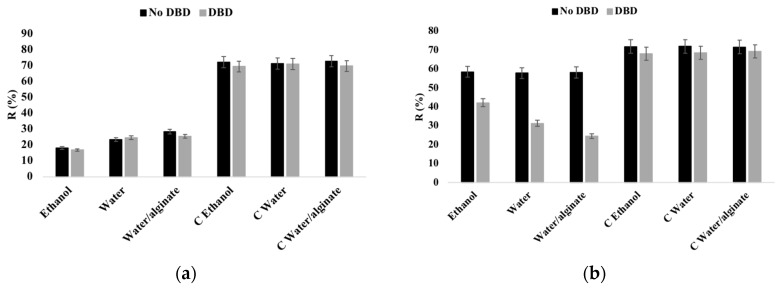
Reflectance (%) at 420 nm of the untreated and dielectric barrier discharge plasma (DBD-plasma) treated samples with PVP-AgNPs deposited by (**a**) exhaustion at 30 °C and (**b**) spray methods; C—Control samples.

Diffuse reflectance measurements were performed to evaluate the relative amount of AgNPs in PA66 samples (Figure 1). In this work, diffuse reflectance can be influenced by two factors: the roughness surface of the samples and AgNPs content. DBD plasma treatment is a surface treatment able to modify the top-most layers of textile surfaces, increasing the surface roughness without altering the bulk material, which decreases the reflectance values and also improves the mass transfer properties [35,36]. Additionally, the AgNPs on the fabric samples are able to absorb light in the visible spectra. Consequently, a high concentration of AgNPs on the fabrics will decrease the reflectance values [37]. Since the characteristic peak of the 20 nm AgNPs appears at around 420 nm, this wavelength was considered for its detection [38].

**Figure 2:**
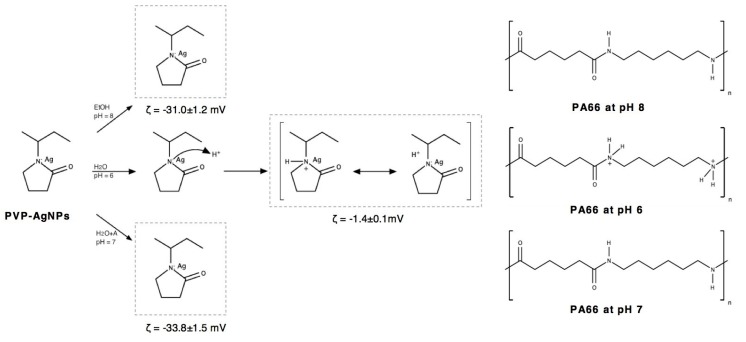
Scheme illustrating the possible chemical interactions between PVP-AgNPs in ethanol, water and water/alginate dispersions and polyamide 6,6 fabric (PA66) surface.

**Figure 3:**
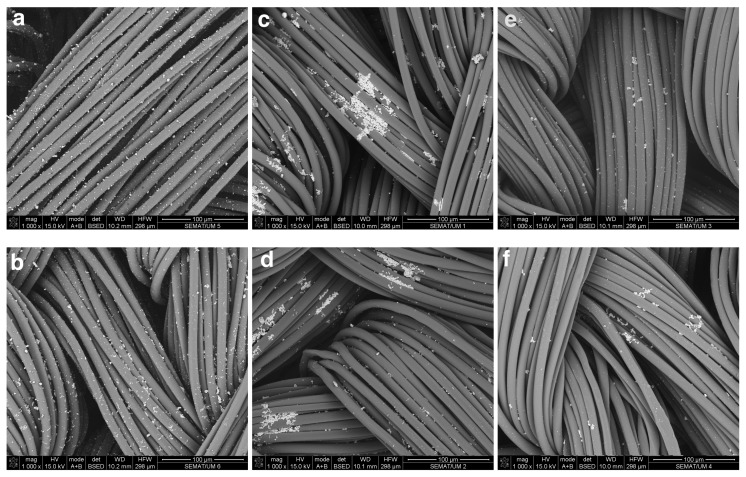
Scanning Electron Microscopy (SEM) images of untreated and DBD-plasma-treated PA66 samples with PVP-AgNPs deposited by exhaustion method at 30 °C with ethanol (**a,b**), water (**c,d**) or water/alginate (**e,f**) as solvents, with a magnification of 1000x.

**Figure 4:**
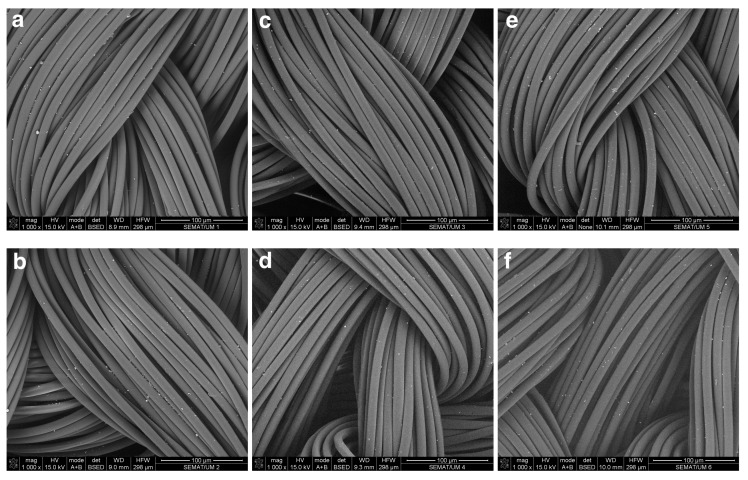
SEM images of untreated and DBD-plasma-treated PA66 samples with PVP-AgNPs deposited by spray method with ethanol (**a,b**), water (**c,d**) or water/alginate (**e,f**) as solvents, with a magnification of 1000x.

Studying the exhaustion method at 30 °C, the lowest reflectance was observed in ethanol dispersions, followed by water and finally water/alginate samples, suggesting a higher amount of nanoparticles deposited in the ethanol samples, which is in agreement with the SEM images (Figures 3 and 4) and relative chemical composition in XPS analysis (Table 2), presented below. In the exhaustion method, no significant difference was observed between untreated and DBD-treated samples. It is well known that the plasma modifications on the fabric surface are not permanent. After a period of time, the surface returns to its original state (ageing) due to the reorganization of the oxidized species and functional groups into the polymer bulk [39]. The DBD plasma effect was only relevant in spray samples showing remarkable differences in reflectance among ethanol, water and water/alginate dispersions. The best result in the spray samples was obtained with the use of the water/alginate dispersion, followed by water dispersion. No differences or decrease in reflectance were observed in the spray-untreated samples using different solvents. It is clear that the solvent has a critical role in the PVP-AgNPs adhesion and distribution performances onto the fabric surface. Hydrophobic interactions between PVP and PA66 alkyl chains can be improved by the use of PVP-AgNPs dispersed in the amphiphilic ethanol solvent. This seems to reduce the existing surface charge repulsions observed in water samples displaying an apparent negative charge in alkaline conditions (pH = 8) [40,41,42]. At this pH and negative zeta potential (~30 mV), only the nanoparticles are charged (Figure 2). The amine groups from PA66 are not protonated, reducing the charge repulsion effect. Differently, in the AgNPs water dispersion (pH = 6), some amine groups in PA66 are protonated and PVP molecules can be protonated due to the basic character of its nitrogen atom, causing charge repulsions between nanoparticles and the substrate. After the addition of alginate into PVP-AgNPs water dispersions, the pH increases to 7, leading to the same conditions observed in ethanol. However, the interactions are partially hindered by the repulsion between PA66 and water.

DBD plasma treatment activates the fabric surface by the introduction of new polar functional groups. These polar groups are able to increase the surface energy and alter the wettability properties. It can also promote etching and cross-linking, without deteriorating the bulk properties of the material because these effects are only promoted onto the material’s surface [43]. The observed difference in reflectance spectra between the exhaustion and spray methods can be attributed to the plasma major drawback, the ageing effect referred above (Figure 1). This process is greatly accelerated by the immersion of the treated fabrics in a solvent as previously observed by submitting textiles with immobilized nanoparticles with and without DBD plasma treatment at several washing cycles [37]. Thus, the differences between the exhaustion and spray method can be explained by this phenomenon since the application of nanoparticles by spray acts only on the PA66 surface, allowing preferential and direct contact with dispersions with a high ratio of PVP-AgNPs without ageing effect.

PVP-AgNPs can be deposited on different parts of the textile substrate, which are mechanically locked by attractive forces such as Van der Waals or electrostatic ones. Moreover, they can also be attached by chemical interactions such as ionic, metallic and hydrogen bonds [44]. The good results obtained for PVP-AgNPs dispersed in water/alginate revealed the complex chemical nature of the interactions among silver and alginate involving several changes in the Ag oxidation state since the binding of AgNPs by alginate is usually achieved by Ag–O bonding [45]. Alginate has hydroxyl and carboxyl groups that can electrostatically stabilize AgNPs-forming complexes [46].

### 3.3. XPS

PA66 samples with PVP-AgNPs obtained from water and ethanol dispersions were chemically evaluated and compared with the respective control samples using the XPS technique. The relative chemical composition and atomic ratios (O/C and N/C) of untreated and DBD plasma-treated samples were calculated (Table 2). Samples with DBD plasma treatment showed a significant increase in oxygen content and O/C ratio, confirming the incorporation of oxygen species during the treatment at atmospheric pressure in air. The generation of new polar oxygen groups onto the fibers surface promotes its chemical etching and the modification of the surface wettability and adhesion properties [47]. The silver content in samples was considerably different depending on the deposition method. For samples obtained by exhaustion at 30 °C, a superior amount of silver was observed with remarkable difference between ethanol and water, but also among untreated and DBD plasma-treated samples; 3.5% and 2.3% for ethanol, and 1.1% and 1.5% for water, respectively, confirming the superior attraction forces between ethanol and PA66. The sprayed samples presented a lower amount of silver compared with the exhaustion method. In this case, water-dispersion without DBD plasma treatment (0.7%) presented more silver on the PA66 surface than DBD (0.3%). It is important to highlight that the relative composition of silver, considering the XPS results, indicates the surface content (sampling depth is commonly less than 10 nm) [48]. Comparing the XPS and reflectance analysis of this sample, it indicates a superior PVP-AgNPs concentration inside the fiber in DBD plasma-treated sample but an inferior concentration onto its surface. Despite being less obvious, the same situation was observed using ethanol dispersion in the exhaustion method samples. Using water dispersions and exhaustion method, the DBD plasma treatment slightly improved the PVP-AgNPs adhesion on the PA66 surface (without DBD 1.4% and with DBD 1.5%).

The chemical modifications of PA66 surface by DBD plasma treatment and the chemical bonding information were obtained by the deconvolution spectra of C1s, N1s, O1s, and Ag3d (Table 3). The C1s envelopes of untreated samples and ethanol DBD plasma-treated sample obtained from exhaustion method can be fitted in three peaks at 285.0, 286.2 and 287.8 eV (Figure S1 in Supplementary Materials). The main peak at 285.0 eV was attributed to C–C and C–H bonds. The peak assigned to C–N and O=C–N bonds emerged at 286.2 eV and 287.8 eV, respectively [36]. The remaining DBD plasma-treated samples presented the same peaks and a new fourth peak at 289.1 eV attributed to O–C=O bonds, that prove the introduction of new oxygen components in the PA66 surface by DBD plasma treatment, which are typically associated to anhydride, carboxyl and ester groups [49]. The presence of new polar functional groups may be attributed to bond scission or the oxidation of methylene groups on the PA66 surface with an increase in PA66 surface energy [50]. The O1s spectra presented two peaks attributed to O=C (531.6 eV) and O–C bonds (533.1 eV). The peak at 531.6 eV was predominant in all samples but, after DBD plasma-treatment, this peak decreased (Figure S2 in Supplementary Materials). After plasma, the peak intensity of oxygen single bonded to carbon atoms (533.1 eV) increased in agreement with the C1s deconvolution analysis. This suggests a strong PA66 surface oxidation. The N1s region deconvolution resulted in two peaks at 399.7 and 401.6 eV, which are attributed to N–C and N–H, respectively (Figure S3 in Supplementary Materials) [51,52]. The Ag3d XPS spectra of PA66 samples were performed to further identify the oxidation states of AgNPs in the different tested conditions. The silver peaks analysis showed two doublets (Figure S4 in Supplementary Materials). The main doublet was attributed to metal silver and appeared at 368.3 (Ag 3d5/2) and 374.3 eV (Ag 3d3/2) [53]. The second doublets at 369.6 eV and 375.7 eV were attributed to ionic silver formed on the surface of AgNPs [54]. The intensity of the second doublet was superior in all DBD plasma-treated samples, indicating the influence of the treatment in the AgNPs oxidation.

### 3.4. SEM

The SEM images analysis confirm the significant differences previously observed in the reflectance and XPS analysis among the used solvents, the plasma treatment and the different deposition methods (Figure 3 and Figure 4). In the exhaustion method, the differences among the three used dispersions are noticeable. The ethanol dispersion showed an even deposition onto the fiber’s surface with few nanoparticle aggregations due to the interactions among the amphiphilic PVP-AgNPs, solvent and PA66 surface as previously discussed (Figure 3a). The water and water/alginate dispersions (Figure 3c,e) are characterized by the presence of numerous PVP-AgNPs aggregations with low deposition, especially with water/alginate that also shows a very low amount of PVP-AgNPs onto the fiber surfaces. The DBD-treated fabrics did not show significant differences with the untreated samples, maintaining the same patterns among the used solvents (Figure 3b,d,f) due to the ageing effect. The exhaustion method is very efficient in PVP-AgNPs deposition using ethanol without the need for DBD plasma to aid the NPs deposition.

Different to the exhaustion method, the spray application of the PVP-AgNPs onto the fibers is characterized by lower deposition efficiency with no observable differences between DBD-treated and untreated samples. Despite the apparent lower amount of deposited PVP-AgNPs compared to the exhaustion method, the reflectance spectra and the antimicrobial efficiency showed similar results for DBD-treated water samples. This could be related to the small dimension of the single nanoparticle and the absence of noticeable aggregates that could be below the detection limits at the used magnification.

### 3.5. Antimicrobial Activity

**Figure 5:**
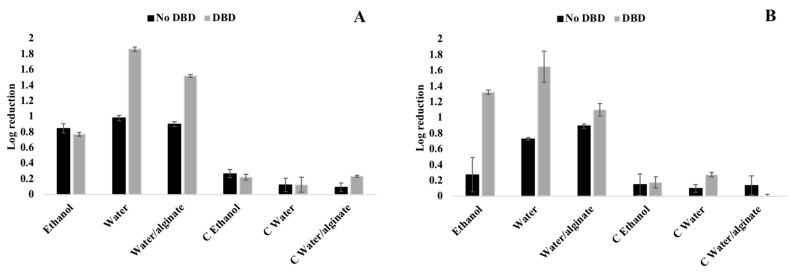
*S. aureus* reduction of the untreated and DBD-plasma-treated samples with PVP-AgNPs deposited by exhaustion at 30 °C (**A**) and spray (**B**) methods; C—Control samples.

**Figure 6:**
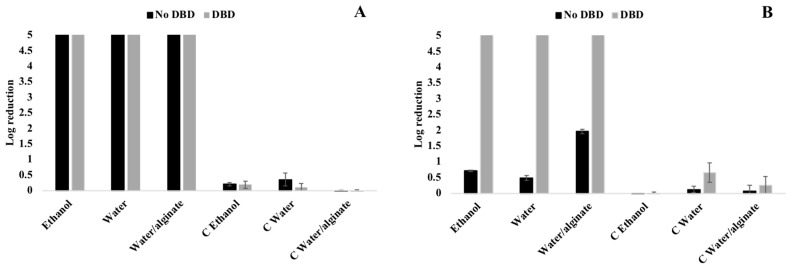
*E. coli* reduction of the untreated and DBD-plasma-treated samples with PVP-AgNPs deposited by exhaustion at 30 °C (**A**) and spray (**B**) methods; C—Control samples.

The antimicrobial activity of AgNPs is related to different factors such as size, shape, concentration, surface charge, and tolerance of bacteria to nanoparticles [55]. In this study, there are significant differences in the antimicrobial efficiency of samples obtained by exhaustion and spray methods for both Gram-positive and Gram-negative bacteria (Figure 5 and Figure 6). On one hand, the activity of the Gram-positive bacteria *S. aureus* is generally lower than the Gram-negative *E. coli*. On the other hand, the exhaustion method displays similar or higher antimicrobial activities compared to the spray method, which is mostly related with the AgNPs amount on the fabrics surface. However, when DBD plasma is applied, the spray method shows similar or in some cases better antimicrobial effect than the exhaustion method, due to the higher local PVP-AgNPs surface concentration and PVP-AgNPs oxidation state.

DBD plasma treatment shows a general increase in antimicrobial activity for *S. aureus,* especially for water and water/alginate-suspended nanoparticles (Figure 5). It has been previously proved that the Ag^+^ ions released from the AgNPs surface are really responsible for the AgNPs antimicrobial activity destabilizing the cell membrane and the respiratory chain of bacteria [56]. DBD plasma treatment is able to generate new reactive oxygen species that easily oxidize AgNPs, changing the nature and kinetic of the Ag^+^ ions’ release from NPs surface [37]. Furthermore, the roughness created by DBD plasma treatment onto the fiber’s surface improves bacteria adhesion and thus their interactions with the immobilized nanoparticles. It is important to notice that the deposition of PVP-AgNPs dispersed in ethanol using the exhaustion method did not show significant differences between treated and untreated plasma samples (Figure 5A). The superior reduction strength of ethanol compared to water allowed it to react for a longer time with the DBD polar-formed species due to the lack of evaporation in the exhaustion conditions, chemically reducing the fiber surface and suppressing the DBD effect. This effect is not noticed in the water-based suspensions due to the water’s higher oxidation potential.

Excellent antimicrobial activity is observed for Gram-negative bacteria *E. coli* in the exhaustion method samples with no differences between untreated and plasma treated samples (Figure 6A). However, when the PVP-AgNPs are applied by spray, only the DBD plasma-treated samples show total inhibition for *E. coli* (Figure 6B). The sprayed AgNPs are deposited almost exclusively on the surface of the fabric fibers. Independently to the plasma treatment, the fabric has no affinity to the sprayed nanoparticles due to the lack of a noticeable mass-transfer effect as provided by the exhaustion method.

The better performance of PVP-AgNPs in *E. coli* compared to the *S. aureus* samples can be explained by the cell walls’ structural differences between the two types of bacteria. The Gram-positive thick peptidoglycan layer is more difficult to destabilize than the thinner, but has a more complex structure than the Gram-negative cell-wall, suggesting that the antimicrobial activity is regulated by the ability of the Ag^+^ ions to destabilize the bacterial cell-wall [57].

## 4. Conclusions

Several concerns about nanoparticles’ toxicity have emerged during the last years. One efficient strategy to avoid these drawbacks, enhancing antimicrobial efficacy and reducing cytotoxicity, can be the concomitant application of three conditions: (i) decrease of the AgNPs concentration, (ii) reduce agglomeration, and (iii) improve the bonding between AgNPs and textile substrates. In this work, the DBD plasma treatment, beyond its capability to improve adhesion and change the wetting properties of a material, also improved the AgNPs antimicrobial properties by altering its oxidation state. Using spray, it was possible to reduce the AgNPs concentration and agglomeration onto the fabrics; and using DBD plasma treatment, a low-cost and environmentally-friendly technology, it was possible to keep a suitable antimicrobial action.

Antimicrobial efficacy of PVP-AgNPs onto untreated and DBD plasma-treated PA66 samples were tested considering its deposition by spray and exhaustion at 30 °C methods using dispersions in ethanol, water and water/alginate. In the exhaustion method, the PVP-AgNPs deposition efficacy showed to be directly related to the attractive forces and charge interactions between the dispersion solvent, PVP-AgNPs and PA66 fabric. Using ethanol dispersions, the hydrophobic interactions between PVP-AgNPs and PA66 alkyl chains decreased due to its amphiphilic character. Additionally, the pH variations can affect the PVP-AgNPs and PA66 fabric charge. In ethanol (pH 8), the nanoparticles are negatively charged and the amine groups from PA66 are not protonated, which reduces the charge repulsion effect. The DBD plasma treatment was not noticed in reflectance and SEM analysis of samples obtained by the exhaustion method; this suggested an accelerated ageing effect when PA66 fabrics are immersed in the solvent. In spray samples, the ageing effect did not occur and DBD plasma-treated samples showed a superior performance. Antimicrobial activity showed to be improved when more oxidizing conditions were available. DBD plasma treatment showed a general increase in antimicrobial activity especially for *S. aureus*, even if a lower PVP-AgNPs was detected. Despite the superior PVP-AgNPs uniformity deposition using the ethanol, the water’s higher oxidation potential showed to improve the antimicrobial effect.

## Figures and Tables

**Table 1:** Size, ζ potential and polydispersity index (PdI) values of Polyvinylpyrrolidone-coated silver nanoparticles (PVP-AgNPs) dispersions in ethanol, water and water/alginate solutions.

PVP-AgNPs Dispersion	Size (d.nm)	ζ (mV)	PdI
Ethanol	281.0 ± 1.5	−31.0 ± 1.2	0.10 ± 0.02
Water	188.0 ± 0.7	−1.40 ± 0.1	0.30 ± 0.02
Water/Alginate	156.6 ± 0.5	−33.8 ± 1.5	0.40 ± 0.01

**Table 2:** Relative chemical composition of untreated (UT) and DBD plasma-treated (DBD) PA66 samples with PVP-AgNPs provided from water (W) and ethanol (E) dispersions by spray (Sp) and exhaustion (Ex) methods.

Solvent	Samples	C (At%)	O (At%)	N (At%)	Ag (At%)	O/C ratio	N/C ratio
Water	Sp UT	81.98	9.20	8.19	0.68	0.11	0.10
	Sp DBD	81.05	10.64	8.03	0.28	0.13	0.10
	Ex UT	81.13	9.35	8.12	1.40	0.12	0.10
	Ex DBD	80.58	11.29	6.59	1.54	0.14	0.08
Ethanol	Sp UT	82.41	9.05	8.31	0.23	0.11	0.10
	Sp DBD	80.71	11.18	7.85	0.26	0.14	0.10
	Ex UT	84.48	6.83	5.20	3.49	0.08	0.06
	Ex DBD	81.60	10.19	5.91	2.30	0.12	0.07

**Table 3:** Deconvolution analysis of the C1s, O1s, N1s, and Ag3d peaks for the untreated (UT) and DBD plasma-treated (DBD) PA66 samples (SD ± 0.3 eV).

Solvent	Sample	C1s				O1s		N1s		Ag3d			
		368.3 eV	286.2 eV	287.8 eV	289.1 eV	531.6 eV	533.1 eV	399.7 eV	401.3 eV	368.3 eV	369.6 eV	374.3 eV	375.7 eV
Water	Sp UT	48.6	16.9	14.3	-	75.6	24.4	91.7	8.3	48.6	10.8	33.3	7.3
	Sp DBD	47.6	18.0	15.2	1.3	67.3	32.7	89.3	10.7	47.6	9.6	33.9	8.9
	Ex UT	49.4	15.3	13.9	-	77.1	22.9	44.6	55.4	49.4	10.5	32.9	7.3
	Ex DBD	45.1	22.5	14.9	3.3	65.8	34.2	84.4	15.6	45.1	14.3	33.2	7.4
Ethanol	Sp UT	52.1	19.3	13.0	-	82.3	17.7	89.1	10.9	52.1	11.9	28.1	7.9
	Sp DBD	47.9	16.8	13.6	1.7	75.0	25.0	85.2	14.8	47.9	14.2	25.0	13.0
	Ex UT	54.8	13.2	14.8	-	84.9	15.1	83.9	16.1	54.8	6.8	33.0	5.4
	Ex DBD	44.4	15.0	13.1	-	72.8	27.2	85.7	14.3	44.4	14.6	31.1	9.9

## Supplementary Materials

The following are available online at https://www.mdpi.com/2079-4991/10/4/607/s1, Figure S1: High-resolution deconvoluted XPS spectra of the C1s binding energy region of untreated and DBD plasma-treated PA66 samples with PVP-AgNPs in water (W) and ethanol (E) dispersions deposited by spray (Sp) and exhaustion (Ex) methods, Figure S2: High-resolution deconvoluted XPS spectra of the O1s binding energy region of untreated and DBD plasma-treated PA66 samples with PVP-AgNPs in water (W) and ethanol (E) dispersions deposited by spray (Sp) and exhaustion (Ex) methods, Figure S3: High-resolution deconvoluted XPS spectra of the N1s binding energy region of untreated and DBD plasma-treated PA66 samples with PVP-AgNPs in water (W) and ethanol (E) dispersions deposited by spray (Sp) and exhaustion (Ex) methods, Figure S4: High-resolution deconvoluted XPS spectra of the Ag3d binding energy region of untreated and DBD plasma-treated PA66 samples with PVP-AgNPs in water (W) and ethanol (E) dispersions deposited by spray (Sp) and exhaustion (Ex) methods, Figure S5: DBD plasma semi-industrial prototype machine (Softal GmbH/University of Minho, Guimarães, Portugal).
