# Supplementary material for: Effect of Dispersion Solvent on the Deposition of PVP-Silver Nanoparticles onto DBD Plasma-Treated Polyamide 6,6 Fabric and Its Antimicrobial Efficiency
Source: Nanomaterials (Basel). 2020 Mar 26;10(4):607. doi: 10.3390/nano10040607 (PMC7221693; doi:10.3390/nano10040607)
Supplement: Supplementary file 1 [file nanomaterials-10-00607-s001.pdf]

nanomaterials

Supplementary Material

Effect of dispersion solvent on the deposition of PVP-silver nanoparticles onto DBD plasma-treated Polyamide 6,6 fabric and its antimicrobial efficiency

Ana I Ribeiro[1], Martina Modic[2], Uros Cvelbar[2], Gheorghe Dinescu[3], Bogdana Mitu[3], Anton Nikiforov[4], Christophe Leys[4], Irina Kuchakova[4], Mike De Vrieze[5], Helena P. Felgueiras[1], António P Souto[1] and Andrea Zille[1*]

[1]2C2T - Centro de Ciência e Tecnologia Têxtil, Universidade do Minho, Campus de Azurém, 4800-058 Guimarães, Portugal; azille@2c2t.uminho.pt
[2] Jožef Stefan Institute, Ljubljana, Slovenia; uros.cvelbar@guest.arnes.si
[3] National Institute for Lasers, Plasma and Radiation Physics, Ma˘gurele, Romania; mitub@infim.ro
[4] Department of Applied Physics, Ghent University, Ghent, Belgium; anton.nikiforov@ugent.be
[5] Centexbel Ghent, Technologie park 7, 9052, Ghent, Belgium; mike.devrieze@centexbel.be
***Corresponding Author:** Andrea Zille, E-mail: azille@2c2t.uminho.pt

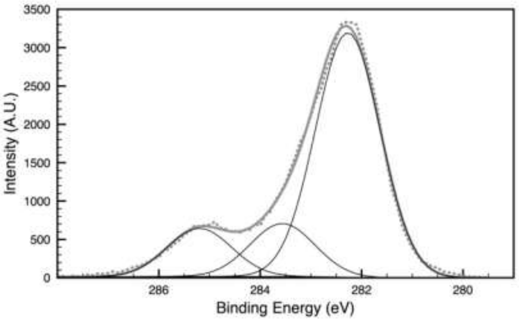

Control exhaustion in water

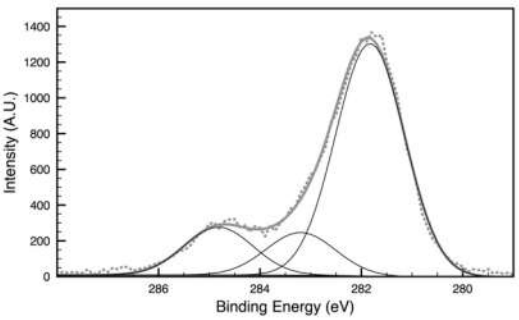

Control exhaustion in ethanol

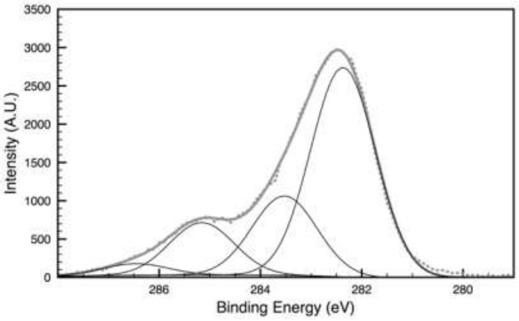

DBD exhaustion in water

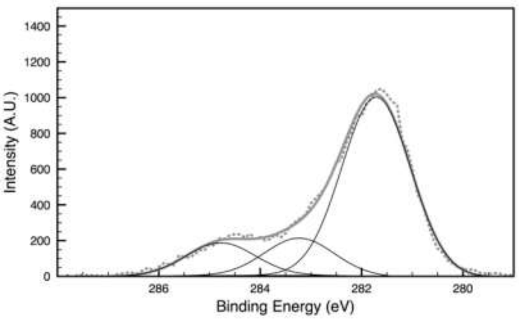

DBD exhaustion in ethanol

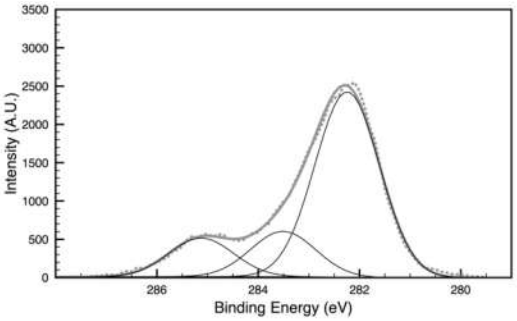

Control spray in water

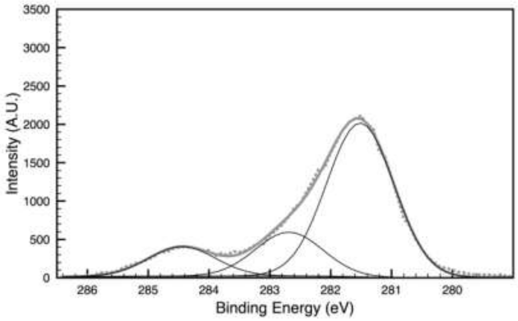

Control spray in ethanol

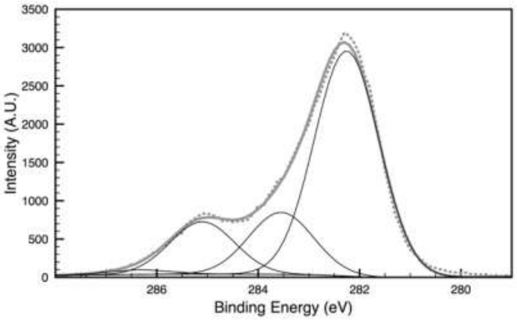

DBD spray in water

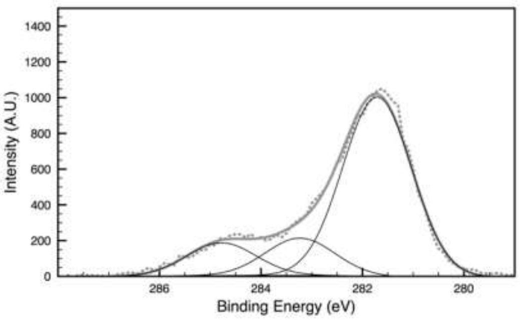

DBD spray in ethanol

Figure S1. - High-resolution deconvoluted XPS spectra of the C1s binding energy region of untreated and DBD plasma-treated PA66 samples with PVP-AgNPs in water (W) and ethanol (E) dispersions deposited by spray (Sp) and exhaustion (Ex) methods.

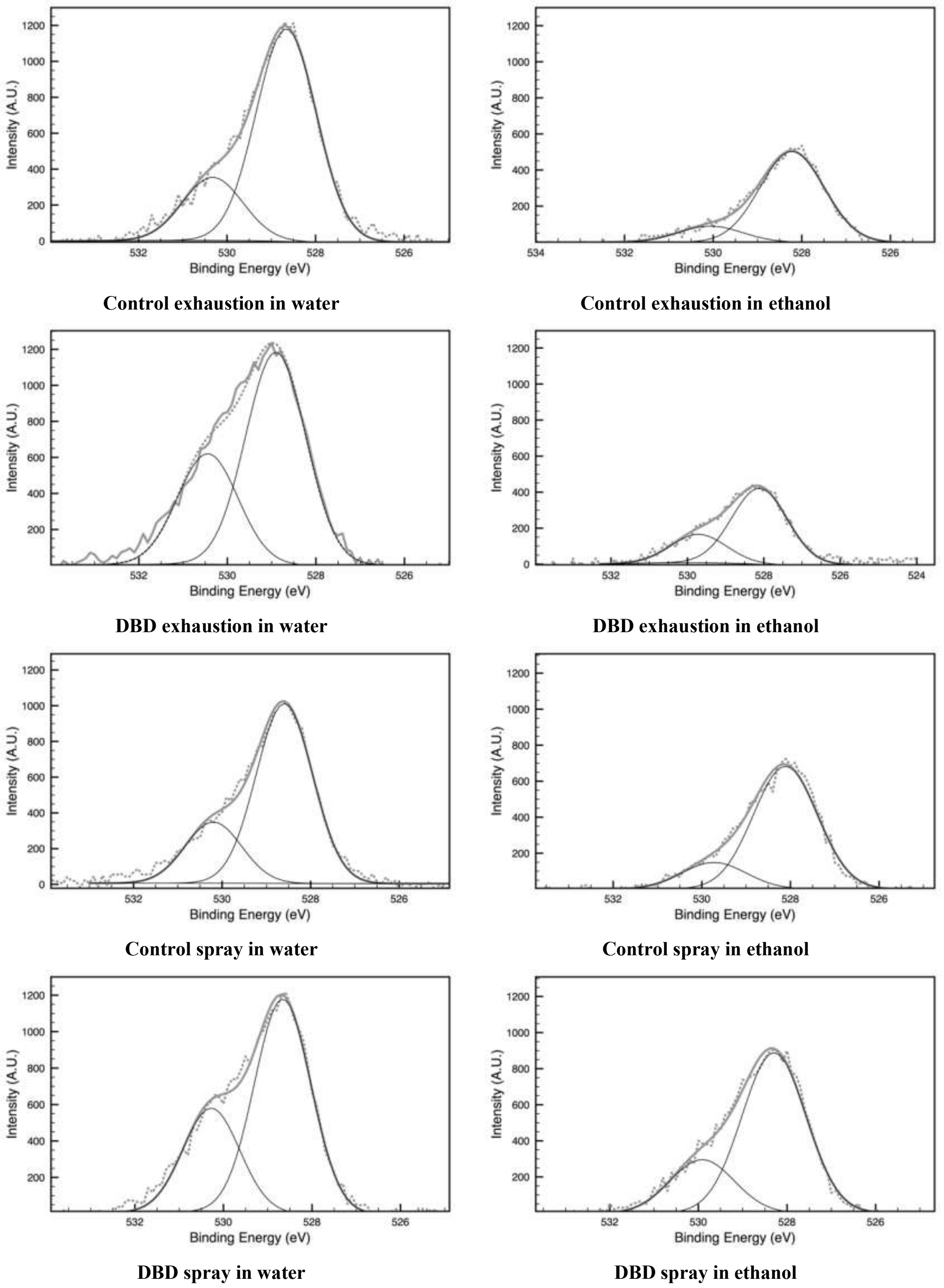

Figure S2. - High-resolution deconvoluted XPS spectra of the O1s binding energy region of untreated and DBD plasma-treated PA66 samples with PVP-AgNPs in water (W) and ethanol (E) dispersions deposited by spray (Sp) and exhaustion (Ex) methods.

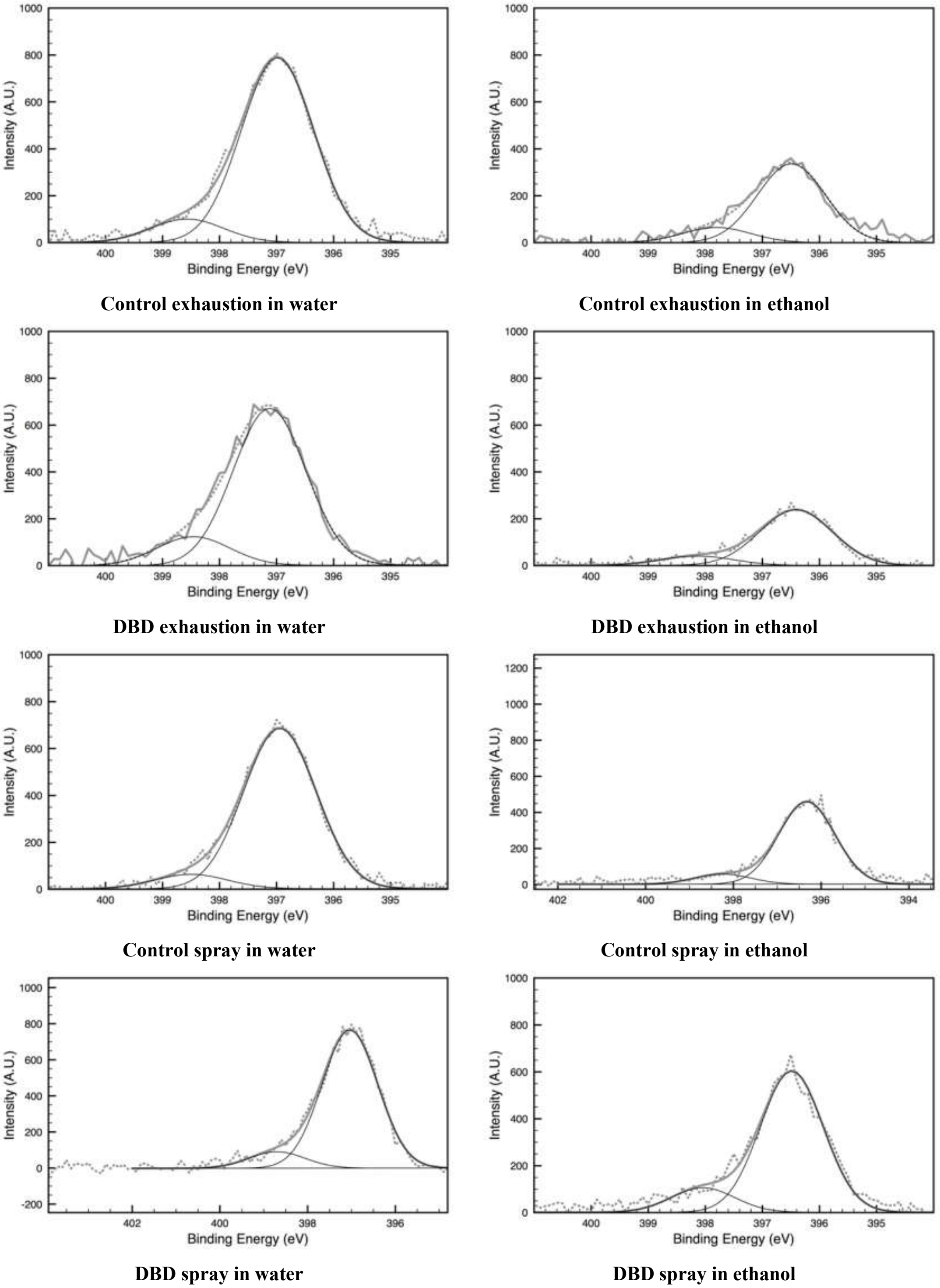

Figure S3. - High-resolution deconvoluted XPS spectra of the N1s binding energy region of untreated and DBD plasma-treated PA66 samples with PVP-AgNPs in water (W) and ethanol (E) dispersions deposited by spray (Sp) and exhaustion (Ex) methods.

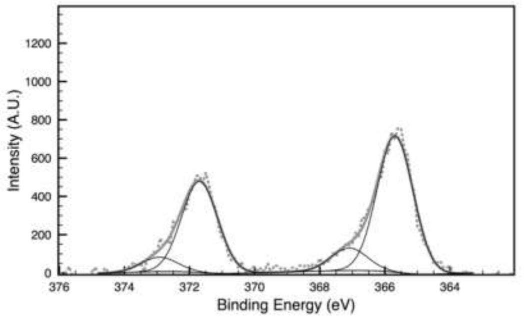

Control exhaustion in water

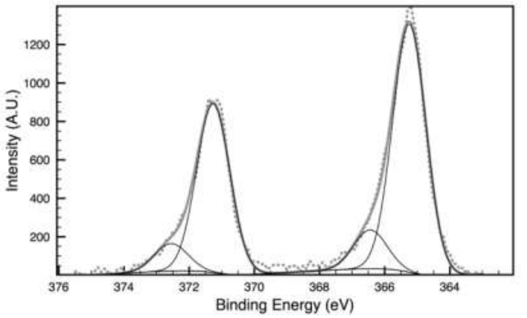

Control exhaustion in ethanol

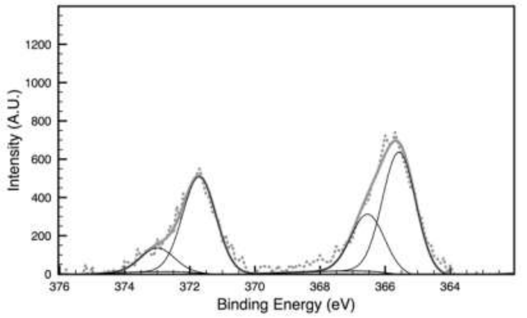

DBD exhaustion in water

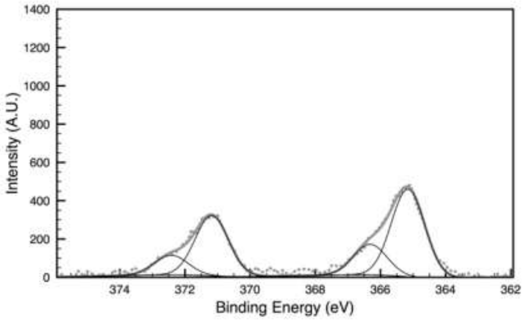

DBD exhaustion in ethanol

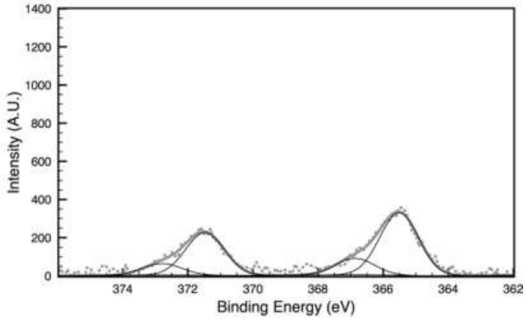

Control spray in water

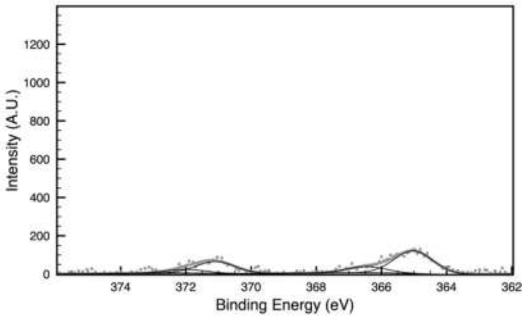

Control spray in ethanol

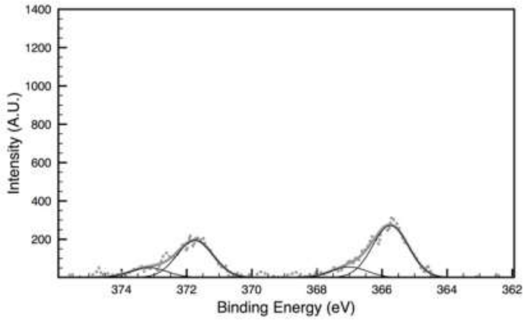

DBD spray in water

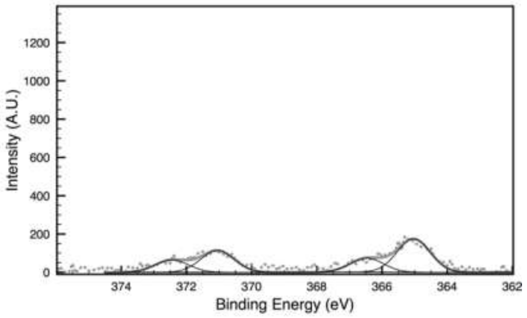

DBD spray in ethanol

Figure S4. - High-resolution deconvoluted XPS spectra of the Ag3d binding energy region of untreated and DBD plasma-treated PA66 samples with PVP-AgNPs in water (W) and ethanol (E) dispersions deposited by spray (Sp) and exhaustion (Ex) methods.

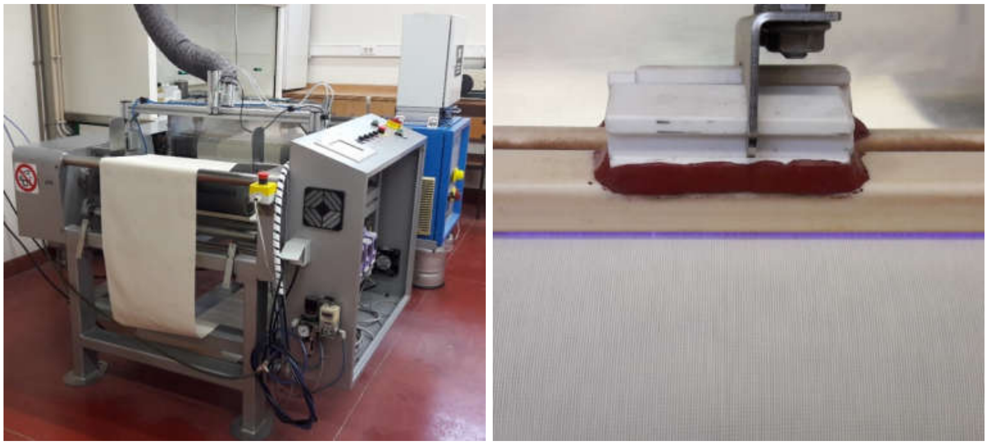

Figure S5. - DBD plasma semi-industrial prototype machine (Softal GmbH/University of Minho, Guimarães, Portugal).
